# Identification of pathogens from blood culture bottles in spiked and clinical samples using matrix-assisted laser desorption ionization time-of-flight mass-spectrometry analysis

**DOI:** 10.1186/1756-0500-7-405

**Published:** 2014-06-27

**Authors:** Simone Konnerth, Gisela Rademacher, Sebastian Suerbaum, Stefan Ziesing, Ludwig Sedlacek, Ralf-Peter Vonberg

**Affiliations:** 1 Institute for Medical Microbiology and Hospital Epidemiology, Hannover Medical School, Carl-Neuberg-Str. 1, D-30625 Hannover, Germany

**Keywords:** Mass spectrometry, Blood culture, Bloodstream infection

## Abstract

**Background:**

Blood stream infections significantly contribute to mortality. An early most appropriate antimicrobial therapy is crucial for a favourable outcome of the patient. Matrix-assisted laser desorption ionization time-of-flight mass spectrometry (MALDI-TOF MS) may speed up the diagnostic of causative micro organisms.

**Findings:**

MALDI-TOF MS using the SARAMIS database was applied to 37 spiked blood culture samples. Identification rates of spiked samples were as follows: The species level was determined in 16 of 21 (76.2%) Gram negative bacteria and in 11 of 13 (84.6%) Gram positive bacteria. Genus level only was determined in additional 2 Gram negative and for the 2 Gram positive strains. Yeast species could not be identified.

MALDI-TOF MS was also compared to cultured-based results in standard routine diagnostic. Identification rates of patient samples were as follows: The species level was determined in 41 of 47 (87.2%) Gram negative bacteria and in 63 of 123 (51.2%) Gram positive bacteria. Genus level only was determined in additional 2 Gram negative bacteria. Once again no yeasts were identified.

A prolonged incubation of BC bottles for 16 hours after primary positive alert did not influence the concentration of bacteria and identification rates.

**Conclusions:**

The SARAMIS database used in our experiments mainly confirms previous findings that were obtained with the MALDI-TOF MS BRUKER system by others.

## Findings

### Background

Blood stream infections significantly contribute to mortality on intensive care units and any delay in appropriate antimicrobial therapy independently increases the risk for a fatal course of disease [1]. That is the reason why the recently published International Guidelines for Management of Severe Sepsis and Septic Shock recommend “early administration of broad-spectrum antimicrobials […], and reassessment of antimicrobial therapy daily for de-escalation, when appropriate” [2].

Changes in antibiotic therapy are mainly based on the microbiological findings from blood cultures (BC). Therefore, rapid identification of pathogens in the laboratory is important to guide treatment of the patient as early as possible. For this purpose, matrix-assisted laser-desorption-ionization time-of-flight mass-spectrometry (MALDI-TOF-MS) applied directly to positive BC broths represents a promising approach [3-8]. In this study we report results from spiked and clinical samples.

### Methods

Blood samples (10 mL each) of a healthy volunteer were spiked with 200 μL of a suspension of microorganisms (McFarland 0.5) and transferred into BC bottles (BACTEC Plus-Aerobic/-Anaerobic, BD, Heidelberg, Germany). Bottles were incubated at 37°C in an automat (BACTEC FX, BD) until reported positive. The same procedure was applied to BC bottles (BACTEC Plus-Aerobic and -Anaerobic and PEDS Plus, BD) from the standard microbiological diagnostic, and results were compared to parallel culture-based testing by certified diagnostic standard operation procedures.

Preparation of pathogen proteins from positive BC bottles was performed as described previously [9]. In brief, sterile syringes were used to transfer 1.5 mL from positive BC bottles into gel-containing tubes (Vacutainer SST II, BD). After centrifuging at 3.000 g at room temperature (RT) for 10 min the supernatant was discarded. The remaining pellet on the surface of the gel was resuspended with 1.3 mL of sterile water. A second centrifugation step in a new sterile tube at 13.000 g at RT was performed for 1 min. Once again, the supernatant was discarded. Of the remaining pellet 0.5 μL were spotted with sterile pipette tips in duplicates (0.5 μL each) onto a FlexiMass MALDI target (Shimadzu Biotech, Basel, Switzerland) for further use.

The mass spectrometer (AXIMA Assurance, Shimadzu) equipped with a 337 nm nitrogen laser and a maximum pulse rate of 50 Hz at 220 V was used for analysis. Spectra were recorded in positive linear mode in the range of 2 to 20 kDa. Data were automatically acquired using the database SARAMIS software 4.09 (AnagnosTec, Potsdam, Germany). Alpha-cyano-4-hydroxycinnamic acid (Alphy Cyano, bioMerieux, Nürtingen, Germany) served as matrix coverage (1 μL). Identification of pathogens was undertaken with and without of 0.5 μL using 25% formic acid. Protein preparation was repeated 16 h after the primary positive alert in order to check for a potential influence of accidentally prolonged incubation of BC bottles.

The study on clinical samples was approved by the institutional ethics committee and the ethics committee waived the need for written informed consent from the blood donor (Ethic Committee Document No. 2074-2013).

### Results and discussion

A total of 37 spiked BC samples (34 bacteria and 3 yeasts) were tested as described. No false positive results were observed. Identification of species was possible for 16/21 (76.2%) Gram negatives, additional 2 (9.5%) strains were correctly identified on genus level only, and the remaining 3 (14.3%) isolates were not identified at all. With respect to Gram positives the species of 11/13 (84.6%) test organisms could be determined by MALDI-TOF-MS directly from the BC bottles, while only genus level was correctly ascertained for the remaining 2 (15.4%). None of the 3 yeasts could be identified in spiked samples in our approach.

A total of 180 BC bottles reported positive from standard microbiological diagnostic were also tested, thereof 120 aerobic, 52 anaerobic, and 8 pediatric BC probes. Parallel subculture on solid media in microbiological diagnostic revealed monomicrobial growth from 161 bottles and polymicrobial growth from 14 BC bottles. The remaining 5 samples did not show growth of any pathogen despite the previous positive alert of the BC automate. MALDI-TOF-MS analysis was then applied to all of the microorganisms from conventional sub-cultivation in order to check for its general ability of identification. The following pathogens were identified by MALDI-TOF-MS after conventional in-between growth on solid media in routine diagnostic processing from BC of patients: *Bacteroides fragilis* (n = 2), *Bacteroides thetaiotaomicron*, *Citrobacter* spp. (n = 2), *Clostridium* spp (n = 3), *Enterococcus faecalis* (n = 8), *Enterococcus faecium* (n = 4), *Enterobacter cloacae*, *Escherichia coli* (n = 22), *Haemophilus influenzae* (n = 3), *Klebsiella oxytoca*, *Klebsiella pneumoniae* (n = 6), *Lactococcus garviae*, *Micrococcus luteus*, *Moraxella osloensis*, *Morganella morganii*, *Pantoea agglomerans*, *Proprionibacterium acnes* (n = 8), *Proteus mirabilis*, *Pseudomonas aeruginosa* (n = 4), *Staphylococcus aureus* (n = 24), *Staphylococcus capitis* (n = 6), *Staphylococcus epidermidis* (n = 39), *Staphylococcus haemolyticus* (n = 7), *Staphylococcus hominis* (n = 13), *Staphylococcus saccharolyticus*, *Stenotrophomonas maltophilia*, *Streptococcus agalactiae*, *Streptococcus mitis* (n = 2), *Streptococcus salivarius*, *Streptococcus* spp. (n = 4), *Candida albicans* (n = 3), and *Candida glabrata* (n = 3). This panel of clinical strains seems quite representative, as the rather large proportion of Gram positive bacteria matches the most frequent pathogens from BC bottles as reported from the German National Reference Centre for the Surveillance of Nosocomial Infections (KISS) [10]. Noteworthy, just like others [11] we were also able to identify several yeasts after grown on solid media by MALDI-TOF-MS analysis.

However, when performed directly from positive BC samples, MALDI-TOF-MS correctly identified the species in 41 (87.2%) and the genus only in 2 (4.3%) of the 47 Gram negatives – among them several species that usually present with highly resistant antimicrobial profiles – compared to 63 (51.2%) identifications on species level of the 123 Gram positives. Once again all yeasts remained unidentified. Identification of yeasts via mass spectrometry relies on rather few peaks only, which may disturb the microbiological diagnostic process [12]. Whether the failure of yeast identification directly from the BC bottles was due to the different growth conditions in the bottle compared to the solid media [13] remains unclear in our study.

**Figure 1:**
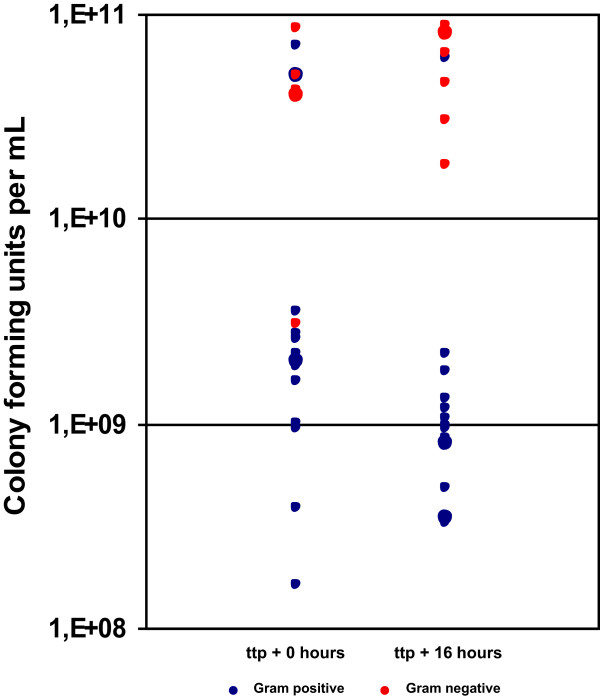
**Comparison of pathogen numbers depending upon incubation time after time to positivity.** Number of colony forming units of micro organisms immediately and 16 hours after positive reporting of the blood culture bottle automate (ttp = time to positivity).

A prolonged incubation of BC bottles after the primary positive alert did not influence the concentration of bacteria as shown in Figure 1. The mean number of colony forming units in the BC bottle at the time point of alert was 1.91 × 10^10^ and 1.89 × 10^10^ at 16 h thereafter respectively. No changes in identification rates were observed either.

At present there is a considerable variation in identification rates of pathogens directly from BC bottles by MALDI-TOF-MS. For example Christner et al. achieved an overall identification rate of 87% [14], while others report difficulties especially in the identification of Gram positive bacteria, fungi and pathogens from polymicrobial samples [15-18]. These discrepant findings may be due to variations in the diagnostic protocol including the BC bottle used [9,19], the preparatory methods for extraction of pathogens [20,21], and the type of mass spectrometer and applied database [8,9,22-24].

Further studies should be undertaken in order to determine optimal conditions for the application of MALDI-TOF-MS technology for the benefit of patients suffering from septicemia [25,26]. Such future approaches may for example focus on limiting the potential loss of pathogens during their extraction from the BC sample. One may also try subtracting interfering background peaks that are due to other patient’s proteins [27]. In addition, a larger number on mass spectrum data – especially of yeasts – could also improve the overall accuracy of species identification by MALDI-TOF-MS analysis.

## Competing interests

The authors declare that they have no competing interest.

## Authors’ contributions

SK: Substantial contribution to conception of the study and performing laboratory work; GR: Substantial contribution to conception and design of the study; SS: Critical revision of the manuscript; SZ: Acquisition of data and technical support; LS: Analysis of data and supervision of the experiments; RPV: Analysis of data and drafting of the manuscript. All named individuals qualify as an author and have given final approval of the version to be published.
